# Development of a quantum dot-based lateral flow immunoassay strip for rapid and sensitive detection of SARS-CoV-2 neutralizing antibodies

**DOI:** 10.1038/s41598-023-49244-5

**Published:** 2023-12-14

**Authors:** Xirong Wang, Shulin Shao, Huan Ye, Sen Li, Bing Gu, Bo Tang

**Affiliations:** 1 Medical Technology School of Xuzhou Medical University, Xuzhou, 221004 China; 2 Department of Laboratory, Nanjing Pukou Hospital of Traditional Chinese Medicine, Nanjing, 211800 China; 3 Nanjing Vazyme Medical Technology Co. Ltd., Nanjing, 210046 China; 4 Nanjing Vazyme Biotechnology Co. Ltd., Nanjing, 210046 China; 5 Laboratory Medicine, Guangdong Provincial People’s Hospital, 106 Zhongshan 2nd Rd, Yuexiu District, Guangzhou, Guangdong 510000 China

**Keywords:** Viral infection, SARS-CoV-2

## Abstract

To a certain extent, the development and vaccination of COVID-19 vaccine have reduced the alarming rate of transmission speed and mortality rate. At present, vaccine coverage is quite high in countries around the world. Since individual differences are unavoidable, it is necessary to assess the efficacy of the vaccine in each vaccinated person in order to reflect the protective effect of the vaccine in different populations. In this study, we developed a novel COVID-19 neutralizing antibody detection kit combining lateral flow immunochromatography and novel quantum dot technology with 85.23% sensitivity, 92.50% specificity. The novel QD-ICA could achieve an accurate detection of SARS-CoV-2 neutralizing antibodies with 10 minutes, two steps, small equipment size, and broad testing application, suggesting its capability to assess vaccine effectiveness on a large scale in areas of world that currently affected by the pandemic.

## Introduction

In December 2019, the coronavirus disease 2019 (COVID-19) was initially identified as being caused by the infection of severe acute respiratory syndrome coronavirus 2 (SARS-CoV-2)^1^. As of 7 October 2022, the global pandemic of novel coronavirus (2019 novel coronavirus, 2019 nCoV) has resulted in more than 618 million (617,597,680) confirmed cases of novel coronavirus pneumonia (COVID-19), with more than 6.5 million (6,532,705) deaths, which has a wide impact on global trade, economic development and social life. At present, a total of 12.7 billion (12,723,216,322) vaccine doses have been administered in the world^2^, intervening in astonishing transmission speed and mortality^3^. However, the effectiveness of available vaccines is still worrying, due to the inevitable relevant individual difference^4,5^. Therefore, it is necessary to evaluate the vaccine effectiveness of each vaccinee to ensure herd immunization^6^. However, there is still a lack of real-time, low-cost, large-scale neutralization antibody detection methods for the evaluation of immune status after vaccine immunization.

SARS-CoV-2 is an enveloped single-stranded positive-chain RNA virus, and is a member of the coronaviridae, β-coronavirus cluster^7^. Spike protein (S) on the surface of virus envelope is a structural protein that mediates virus adhesion and invasion of host cells^8^. S protein is divided into two subunits, S1 and S2. Angiotensin converting enzyme 2 (ACE2) is a receptor of virus invading target cells^9^. S1 combines with ACE2 on host cells through its receptor-binding domain (RBD), initiating S2 conformational change, causing virus-host cell membrane fusion, and promoting virus entering into cells^10^. The binding epitopes of neutralizing antibodies reported are all on S1 protein, and most of them bind to RBD protein^11–14^. Neutralizing antibodies against competitive epitopes of ACE2 will inhibit the binding of RBD to ACE2, thus blocking the virus from entering the host cell^15,16^. Ninety percent of the active neutralizing targets in the serum or plasma of most newly infected and vaccinated individuals are on the RBD^15^. Therefore, it is reasonable to design a COVID-19 neutralization antibody detection method targeting RBD protein.

With the continuous efforts of scientists and vaccine development enterprises, several major vaccines have been provided to the public for the urgent use in vaccination around the world^17^. Reliable and universal serological testing is urgently needed to assess the herd immunity and protective humoral immunity of vaccine candidates. Additionally, asymptomatic patients, suspected cases or reservoirs of COVID-19 can be detected by SARS CoV-2 specific antibody^18^.

In this study, we established a novel COVID-19 neutralizing antibody detection kit, without any cells or live virus, that can be completed in 10 minutes in a BSL-2 laboratory and other application scenarios, e.g., wards, clinics, health centers and families. As in cVNT or pVNT, RBD-ACE2 interaction can be blocked by specific NAbs in patient. In the same way, our test is designed to simulate this virus-host interaction, with purified receptor-binding domain (RBD) from the Spike protein and the host cell receptor ACE2.

## Methods

### Materials and instruments

2-(N-morpholino) ethanesulfonic acid (MES), monoethanolamine and glycine were obtained from Sigma-Aldrich (St. Louis, MO, USA). Sodium hydroxide (NaOH, 96.0%), hydrochloric acid (HCl, 37%), Na_2_HPO_4_·12H_2_O, NaH_2_PO_4_, NaCl, HEPES, Tris, Tween-20, Na_2_CO_3_, NaHCO_3_ and sucrose were purchased from Sangon Ltd (Shanghai, China). N-Hydroxysul-fosuccinimide (sulfo-NHS), N-(3-dimethylaminopropyl)-N′-ethylcarbodiimide hydrochloride (EDC) and bovine serum albumin (BSA) were purchased from Sigma-Aldrich. Streptavidin was purchased from FEBICO. NHS-PEG_4_-Biotin was purchased from Thermo Fisher Scientific. Ultracel-10 regenerated cellulose membrane(Amicon® Ultra) was purchased from Millipore. Dimethyl sulfoxide (DMSO) was purchased from Macklin (Shanghai, China). BCA Protein Quantification Kit, Anti-SARS-CoV-2 Neutralizing Antibody ELISA Kit, RBD recombinant antigen, ACE2 recombinant antigen, POD, POD clon2 and quantum dots were purchased from Vazyme (Nanjing, China). Fluorescence spectra were measured using a microplate reader (Infinite® M200 PRO, TECAN). Real-time dynamic light scattering signals of the QDs and the QDs-RBD probes were detected using a Zetasizer Nano ZS system (Malvern Panalytical, Malvern, UK). Selected QDs-RBD probes were dispensed on the sample pad using a gold-dispensing system (Jinbiao, Shanghai, China). Photoluminescence (PL) spectra of the test (T) line and control (C) line on the QD-ICA strip were recorded using the automatic fluorescence immunoanalyzer QD-S600 (Vazyme). Reagents not mentioned here were all purchased from Vazyme. The ratio of Positive and Negative represented the ratio of the positive standard plasma and negative standard plasma.

### Sample collection

The study was conducted on 208 plasma samples, 110 collected before the outbreak of COVID-19 (from August to October 2019), 98 collected from people who have been vaccinated with COVID-19 vaccine (March 2021). Before the interview and blood collection, written informed consent was obtained. All the pre pandemic samples were tested negative for SARS-CoV-2 S1 RBD antibodies (Supplementary Fig. 1). The samples were divided and frozen at  − 80 °C. Before use, the sample should be placed at room temperature and observed to be completely melted with the naked eye. After centrifugation, the supernatant should be taken. All methods were performed in accordance with relevant guidelines and regulations. The study was approved by the Ethics Committee of Nanjing Drum Tower Hospital.

### Establishment of QDs-RBD conjugates

Quantum dot microspheres and RBD antigen are coupled by biotin-streptavidin system and obtained by mixing QDs-SA and RBD-Biotin vertically at 37 °C for 30 min. QDs-SA conjugates were prepared by conventional EDC/NHS coupling reaction between the amino groups of the SA and carboxyl groups on the hydrophilic surface of QDs. First, 50 μL of QDs (10 mg/mL) was dispersed in 450 μL of MOPS (0.02 M, PH 6.5), followed by activation with 5 μL (50 mg/mL) EDC and 5 μL (75 mg/mL) NHS. The activated QDs on ice were subjected to ultrasound for 5 min and collected by centrifugation at 13000 rpm at 18 °C for 15 min. The QDs sediment was dispersed in 500 µL MES (0.02 M, pH 6.0) and incubated with different amounts of SA for approximately 1 h at room temperature (20 ± 5 °C). Next, we added 1.07 µL (14 mg/mL) of the reference protein POD for quality control and incubated for 1 h at room temperature. The complex was blocked using 5% BSA and terminated using 10% ethanolamine for 30 min at the same temperature. The terminated sediment was obtained by centrifugation and stored in PBS buffer (0.01 M, pH 7.4).

The combination of RBD and biotin is due to the efficient reaction of NHS-activated biotin with primary amine groups (–NH_2_) to form stable amide bonds.0.5 mg RBD and different amounts of biotin (10 mM, dissolved in DMSO) were mixed for 2 h. 50 μL 3% glycine(dissolved in 0.01 M PBS; The solution was prepared just before use) was added to the solution to terminate the reaction. Finally, the labeled solution was transferred to the ultrafiltration tube with 1 × PBS, mixed well, and purified by centrifugation at low temperature for 15 min at 4000 rpm and repeated five times.

### Establishment of optimal proportions of coated ACE2 and QDs‑labeled RBD

To determine the optimal dilution ratios for the QDs-RBD probes and coated antigen ACE2, a checker-board titration test was performed. The coated ACE2 antigen was diluted to 0.5, 1, and 2 mg/mL and sprayed onto T line on NC membrane. QDs-RBD probes were diluted to 0.4, 0.6, and 0.8 mg/mL and sprayed onto conjugate pad. Standard serum (3.2 μg/mL) was added to the sample pad of a QD-ICA strip. As the liquid migrated from the sample pad toward the absorbent pad, QDs-RBD probes were captured at the test line and control line. After reaction for 10 minutes, fluorescence signal on the two lines on NC membrane were recorded by a portable fluorescence reader (365 nm excitation), respectively.

### QD-ICA strip preparation for SARV-CoV-2 neutralizing antibodies detection

**Figure 1:**
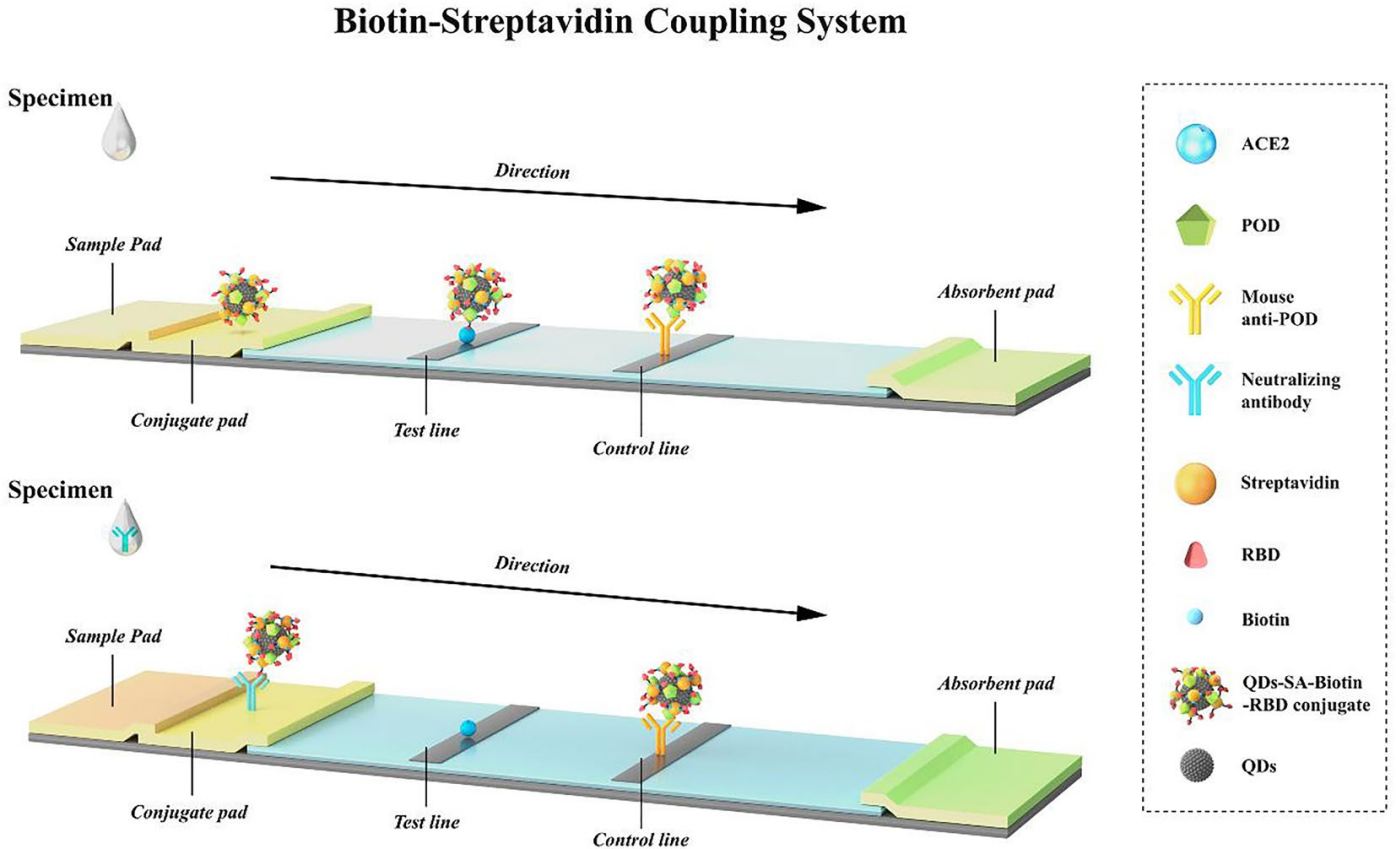
Quantum dot conjugate formation and NAbs detection. Schemes used for identification of SARS-CoV-2 neutralizing antibodies. ACE2 angiotensin converting enzyme 2, RBD receptor-binding domain, QDs quantum dots.

Figure 1 illustrates the QD-ICA for detection of SARV-CoV-2 neutralizing antibodies in human plasma using the SARS-CoV-2 RBD-conjugated QDs.

The QD-based immunochromatographic test strip consists of the sample pad, conjugate pad, absorbent pad, and nitrocellulose (NC) membrane. The test strip was prepared by the competitive method. The steps are as follows. Sample pads (200 × 100 mm) made of glass fiber were placed in Tris buffer (0.05 M, pH 8.5) containing 0.2% casein and 0.12% EDTA-2Na, saturated, and then dried at 60 °C for 2 h. QDs-RBD probes were dispersed in Tris buffer (0.05 M, pH 8.0) containing 20% sucrose solution, 1% Tween 20 and 0.5% casein, sprayed onto the conjugation pad at a volume of 4 μL/cm, followed by drying at 45 °C for 24 h. Recombinant ACE2 and internal reference protein antibodies were sprayed onto NC membranes (0.8 µL/cm) to form T line and C line, respectively, and incubated for 18 h at 37 °C. Above temperature and time have been proven to be better for product performance.

For subsequent detection, all parts were finally assembled and cut into a immunochromatographic strip (length: 3.5 mm; distance between the test and control line is 0.5 cm). Sample diffused forward by capillary force upon addition. Upon passage through the conjugate pad and NC membrane, the neutralizing antibodies in the plasma bind to QDs-RBD and ACE2 antigen, forming a complex. The fluorescence signal was generated by quantum dot excitation and recorded by an automatic QD fluorescence immunoassay analyzer QD-S600 (Vazyme). The level of neutralizing antibodies in the sample is inversely related to fluorescence intensity. After assembling the test strip, several key assay conditions of the QD-ICA, e.g. the coupling ratio, incubation time of the QDs-RBD probes were optimized.

### Analysis of clinical samples via a QD-ICA strip

Human plasma (80 μL) was added onto the sample pad of a QD-ICA strip. RBD protein-conjugated labels were successively captured at the test line and control line as the liquid migrated from the sample pad toward the absorbent pad. After reaction for 10 minutes, fluorescence signal on the two lines were recorded by a portable fluorescence reader (365 nm excitation). The QD-ICA detection was performed thrice for each sample, and the fluorescence data were collected and analyzed.

### Analytical performance of QD-ICA

The inhibition rates tested by QD-ICA correlated well with the results by ELISA (Fig. 8a), although QD-ICA cannot completely replace ELISA, this method can complete the detection in 10 minutes, which is much quicker than ELISA, and also has excellent detection performance. Meanwhile, QD-ICA and ELISA results showed moderate correlation with pVNT results (Fig. 8b and c), indicating that integration of the NAbs IC_50_ tested by pVNT would allow this QD-ICA to determine the immune status of clinical specimens and meet clinical sensitivity requirements.

## Results

### Characterization of QDs‑RBD probes

**Figure 2:**
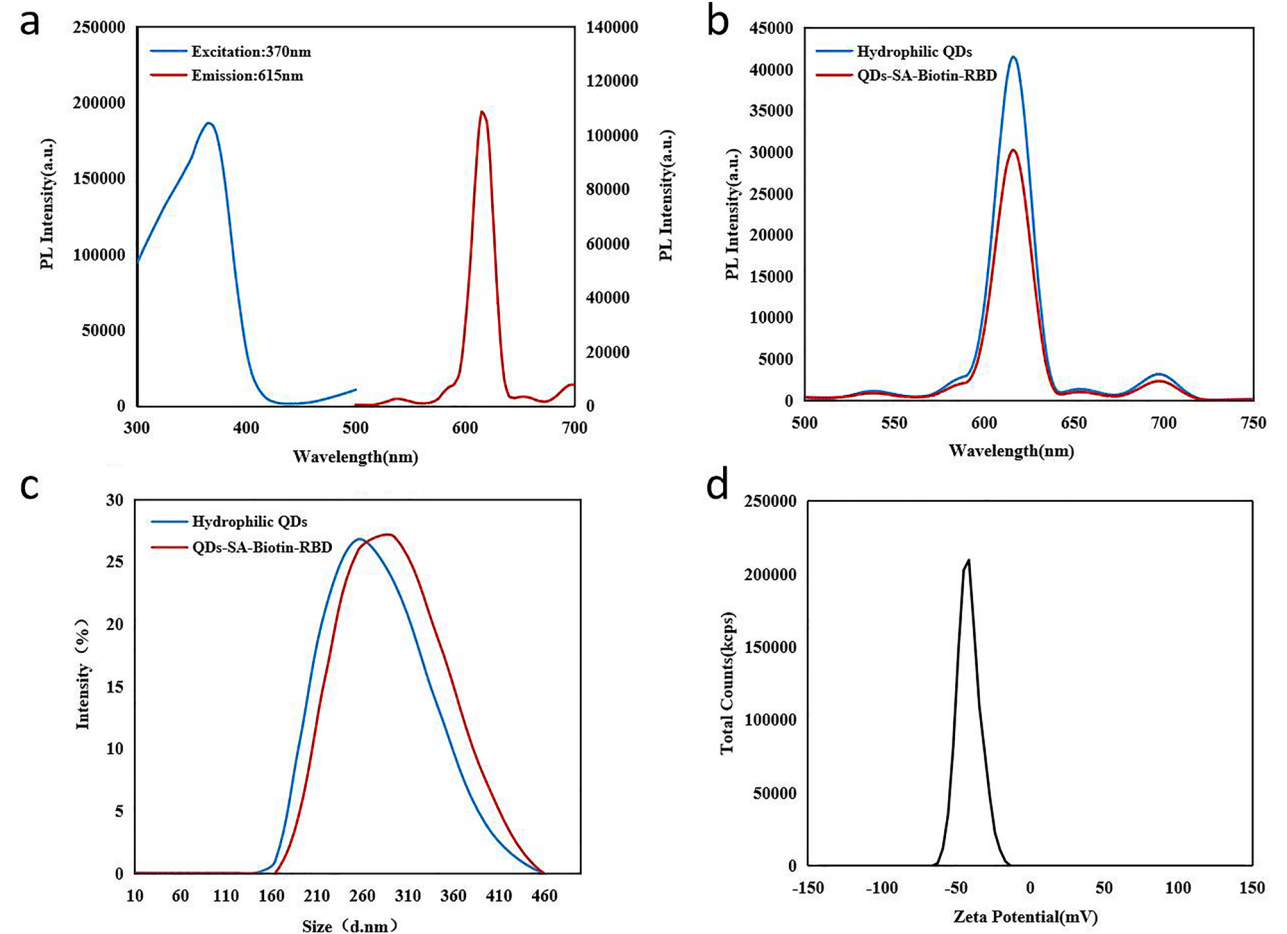
Characteristics of the quantum dot–RBD conjugates. (**a**) Excitation spectra and photoluminescence spectra of the hydrophilic quantum dots. (**b**) Photoluminescence spectra of the hydrophilic QDs and QDs-RBD conjugates. (**c**) Dynamic light scattering of the QDs-RBD conjugates and hydrophilic QDs. (**d**) Zeta potential curves of the QDs-RBD conjugates. PL photoluminescence.

We designed hydrophilic core-shell CdSe/ZnS QDs bound to RBD. We have designed the hydrophilic CdSe/ZnS quantum dots with core-shell structure bound to RBD. In order to change the hydrophobicity of CdSe/ZnS quantum dots and transfer them into aqueous solution, quantum dots were wrapped with amphiphilic polystyrene particles. Figure 2a shows the excitation(370 nm) and photoluminescence spectra (615 nm) of quantum dots wrapped in amphiphilic polystyrene particles.

Polystyrene particles are uniformly bound to the surface of CdSe/ZnS quantum dots. The prepared quantum dots showed narrow particle size distribution and highly uniform monodispersity, with an average particle size of about 260 nm.

**Figure 3:**
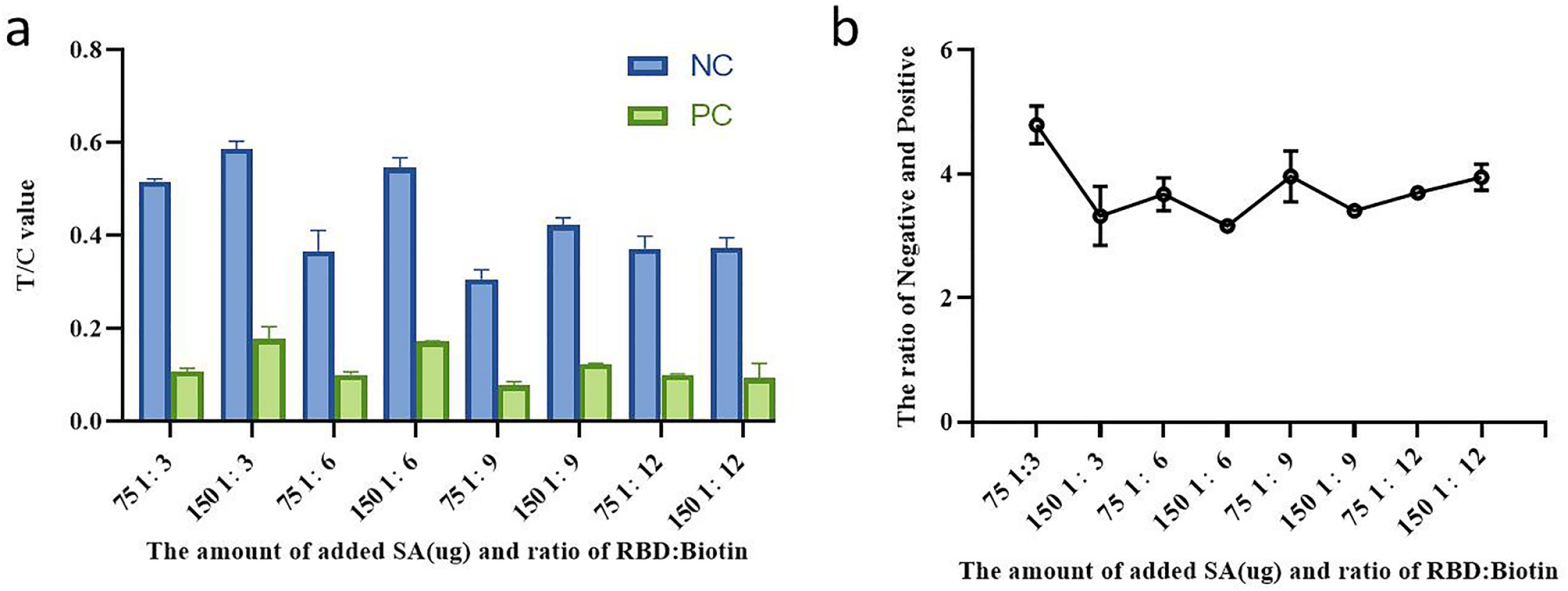
Optimization of the amount of added SA and ratio of RBD:Biotin. The ratio of Negative and Positive represents T/C ratio of healthy control plasma collected before COVID-19 outbreak and positive standard plasma (healthy control plasma mixed with recombinant humanized monoclonal antibody against anti-SRAS-CoV-2 spike protein).

**Figure 4:**
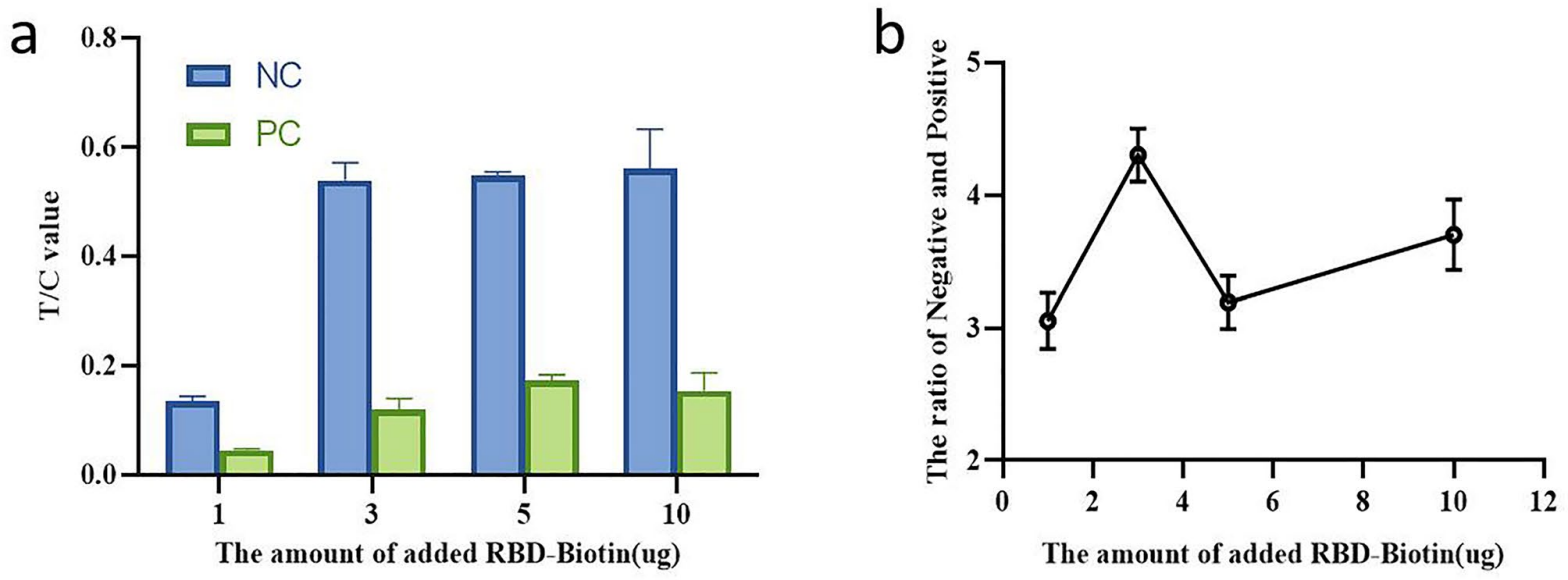
Optimization of the amount of added RBD-Biotin.

**Figure 5:**
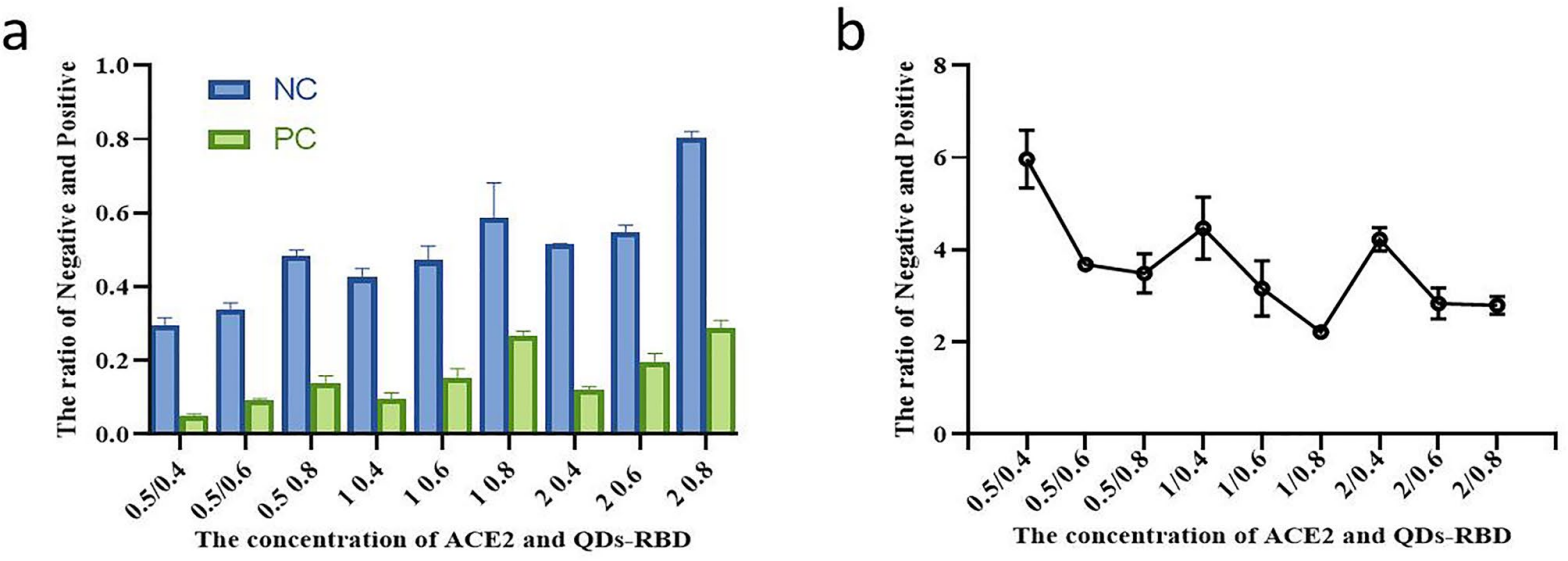
Optimization of the concentrations of ACE2 and QDs-RBD conjugate.

We ascertained the effect of different coupling ratios between QDs and SA, RBD and biotin on the test performance. To be specific, 75 μg SA was used for labeling 100 μL of QDs (10 mg/mL), the coupling ratio of RBD and biotin was 1:3 (Fig. 3). Different amount of RBD-Biotin was added to 100 ul QDs-SA solution to react with negative and positive sample solution, of which 3 μg of RBD-Biotin(1:3) was optimally selected. (Fig. 4). The amount of 0.5 mg/mL ACE2 (4.8 mg/mL) and 0.4 mg/mL anti-POD antibody (14 mg/mL) were optimized for respectively spotting the T line and C line on NC membrane of strip (Fig. 5).

The sizes and fluorescence spectra of hydrophilic CdSe/ZnS quantum dots and QDs-RBD were determined after optimizing the coupling conditions (Fig. 2b and c). There was no significant difference between the fluorescence peak of the QDs-RBD solution and that of the hydrophilic QDs in shape and position, despite a decrease in fluorescence intensity (Fig. 2b). Hydrodynamic analysis after RBD recombinant antigen labeling revealed that the size of the QDs-RBD increased from 255 to 295 nm (Fig. 2c), indicating the successful formation of the QDs-RBD complex. The zeta potential of hydrophilic QDs was then measured to determine the stability. In general, absolute value of zeta potential greater than 30 mV indicates stability in solution^19^. A zeta potential of  − 44.95 mV was determined for the conjugate, indicating that its carboxyl groups are capable of providing enough colloidal stability in aqueous solution (Fig. 2d).

### Optimal proportions of coated ACE2 and QDs-RBD

A checkerboard titration assay was performed to determine optimal dilution ratios of QDs-RBD probes and coated ACE2 concentration. By measuring the ratio of negative and positive of varying concentrations of QDs-RBD and ACE2, we were able to demonstrate that conditions were optimal at a QDs-RBD concentration of 0.4 mg/mL and a ACE2 concentration of 0.5 mg/mL (Fig. 5).

### Determination of incubation time

**Figure 6:**
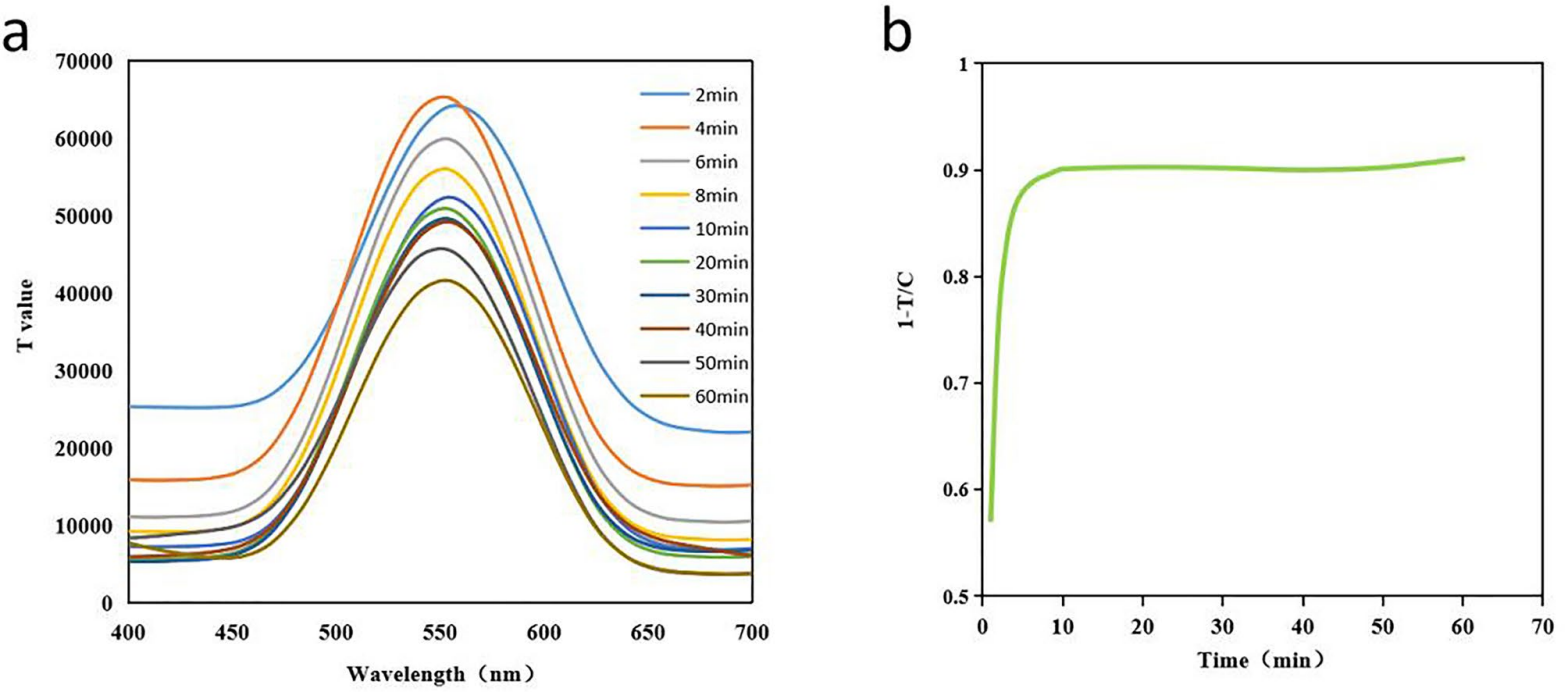
Optimal fluorescence development time for QD-ICA. (**a**) Detection of T value under different incubation times. (**b**) Identification of the optimal incubation time.

We measured fluorescence intensities at different time points to determine the optimal time. We found that the optimal fluorescence development time for QD-ICA to establish dynamic equilibrium between ACE2 and QDs-RBD was 10 minutes (Fig. 6). Temperature and time point stability of quantum dot-based lateral flow immunoassay strip is shown in Supplementary Table 1 and 2. Besides, capability to detect high, medium and low positive samples of QD-ICA along with inter and intra laboratory precision is shown in Supplementary Fig. 2.

### Practical detection of anti-SARS-CoV-2 NAbs in clinical samples

**Figure 7:**
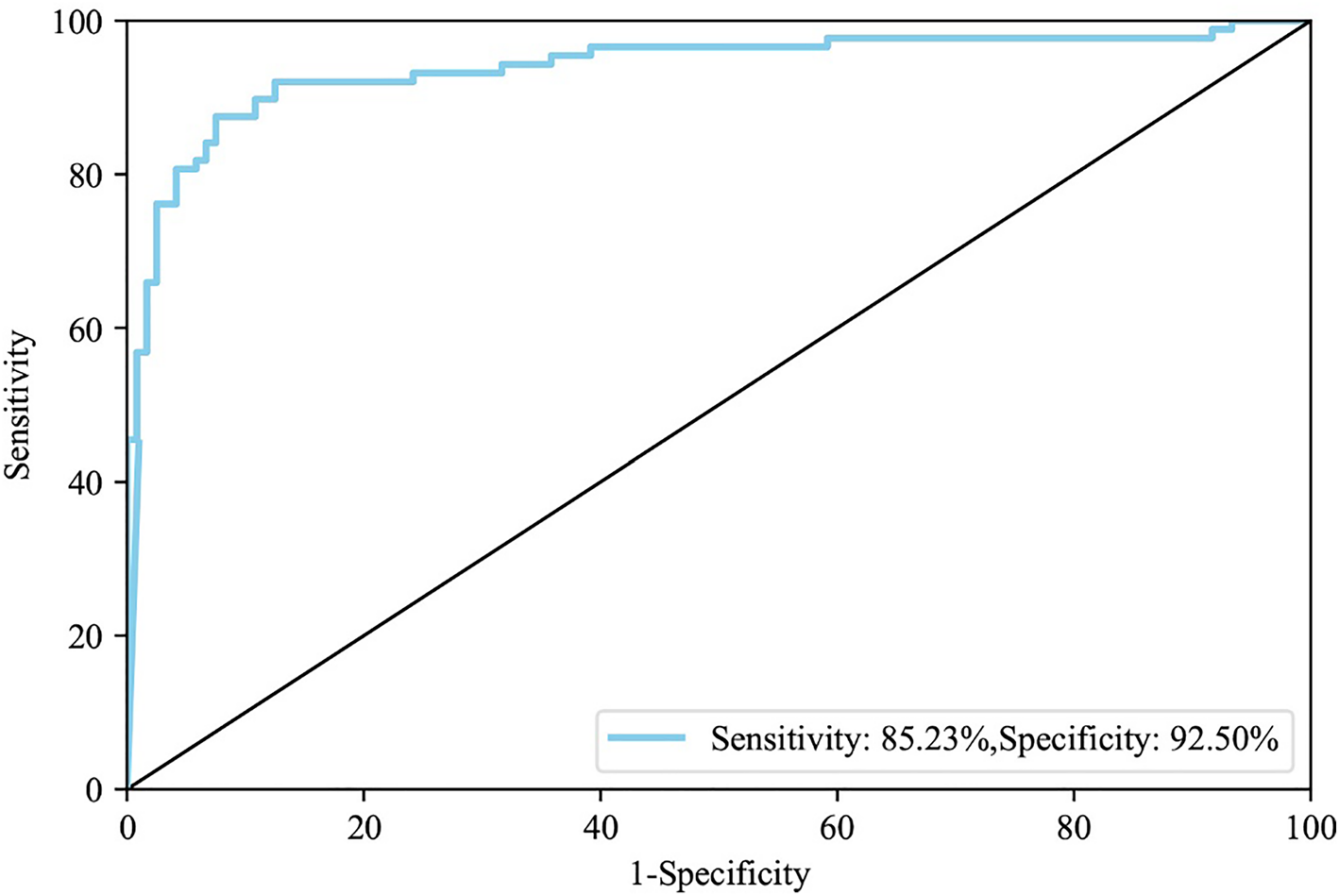
Receiver operating characteristic curve analysis of QD-ICA.

To determine the detection threshold of the CdSe/ZnS QDs-based QD-ICA for detecting anti-SARS-CoV-2 neutralizing antibodies in plasma samples, 110 negative plasma samples and 98 positive plasma samples from vaccinees were measured using this QD-ICA method to obtain the ratio of T/C value, generating ROC curve (Fig. 7).

Correlation and linear regression analyses were performed in Python using R squared and Mean Squared Error. The data presented are the log of the IC_50_ value for pVNT and inhibiton rates of ELISA and QD-ICA. The darker part of the graph represents the 95% confidence interval of the regression equation.

**Figure 8:**
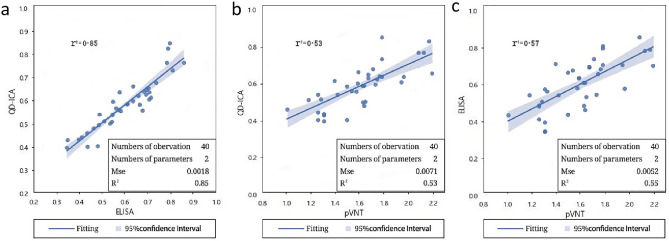
Correlation analysis for samples from 40 vaccinees with different levels of SARS-CoV-2 NAbs by QD-ICA and ELISA (**a**), QD-ICA and pVNT (**b**), and ELISA and pVNT (**c**).

The positive plasma samples were collected from vaccinees with twice vaccination of inactivated vaccine two weeks and eight weeks after the second vaccination, respectively. Based on the QD-ICA results of the negative and positive samples, ROC curves were made and the detection threshold of anti-SARS-CoV-2 NAbs was calculated as 30%. The results of QD-ICA are presented in the form of inhibition rate, and the calculation formula is as follows: Inhibition rate = (1-Sample T/C value /Negative control T/C value) × 100%. In Fig. 8a, the T/C value detected by this method are converted into inhibition rates using this formula. The ELISA results of samples are inversely proportional to the T/C value observed by this method (T line value / C line value) within a certain range. The evident difference in the inhibition rates between negative samples and positive samples demonstrated that the CdSe/ZnS QDs-based QD-ICA method could effectively discriminate whether a plasma sample contains anti-SARS-CoV-2 NAbs and NAbs levels.

## Discussion

In the global 2019-nCoV epidemic, vaccination or prophylactic neutralizing antibodies are effective means to stop the transmission of the virus^20^. The SARS-CoV-2 neutralizing antibody assay based on quantum dot immunochromatography does not require special sample processing, only 80 μL plasma, the NAbs level can be tested within 10 minutes, which can be used for antibody self-assessment and large scale antibody screening, without the need to use live virus and professional technical personnel^21^.

In this study, the SARS-CoV-2 neutralizing antibody detection method was developed based on quantum dot immunoluminescence assay, which showed strong correlation and high consistency with the ELISA results moderate correlation with pVNT results, and has high sensitivity and specificity.

The above results suggest that although the SARS-CoV-2 neutralizing antibody detection assay based on quantum dot immunoluminescence analysis may never be able to completely replace pVNT, our data indicated that the performance of QD-ICA is well correlated with that of both ELISA and pVNT, which demonstrates QD-ICA can be used as an alternative to the gold standard for neutralizing antibody titers in COVID-19 convalescent patients or detection of neutralizing antibodies in people after vaccination, with the advantages of convenience, low cost and easy promotion.

Compared with other existing SARS-CoV-2 neutralizing antibody detection methods^22–24^, the quantum dot immunochromatography method is less expensive and portable, which greatly reduces the overall cost and truly achieves field detection.

In conclusion, the SARS-CoV-2 neutralizing antibody assay developed in this study based on quantum dot immunochromatography can accurately detect the neutralizing antibody level in the sample of COVID-19 convalescent patients and people after vaccination, providing an immediate, efficient and low-cost method for evaluation of population immune status.

### Supplementary Information

Supplementary Information.

## Data Availability

The datasets analyzed in this study can be obtained from the corresponding authors upon reasonable request.

## References

[1] Huang C (2020). Clinical features of patients infected with 2019 novel coronavirus in Wuhan, China. Lancet.

[2] Organization, W.H. *WHO Coronavirus Disease *(*COVID-19*)* Dashboard *[*DB/OL*]. https://covid19.who.int/. Accessed 10 Oct 2022.

[3] Nel AE, Miller JF (2021). Nano-enabled COVID-19 vaccines: Meeting the challenges of durable antibody plus cellular immunity and immune escape. ACS Nano.

[4] Ibarrondo FJ (2021). Primary, recall, and decay kinetics of SARS-CoV-2 vaccine antibody responses. ACS Nano.

[5] Lv H (2020). Cross-reactive antibody response between SARS-CoV-2 and SARS-CoV infections. Cell Rep..

[6] Heaton PM (2021). Herd Immunity: The journey is as important as the destination. J. Infect. Dis..

[7] Lu R (2020). Genomic characterisation and epidemiology of 2019 novel coronavirus: Implications for virus origins and receptor binding. Lancet.

[8] Sun J (2020). COVID-19: Epidemiology, evolution, and cross-disciplinary perspectives. Trends Mol. Med..

[9] Tortorici MA (2019). Structural basis for human coronavirus attachment to sialic acid receptors. Nat. Struct. Mol. Biol..

[10] Jiang S, Hillyer C, Du L (2020). Neutralizing antibodies against SARS-CoV-2 and other human coronaviruses. Trends Immunol..

[11] Wang C (2020). A human monoclonal antibody blocking SARS-CoV-2 infection. Nat. Commun..

[12] Chen X (2020). Human monoclonal antibodies block the binding of SARS-CoV-2 spike protein to angiotensin converting enzyme 2 receptor. Cell Mol. Immunol..

[13] Wu Y (2020). Identification of human single-domain antibodies against SARS-CoV-2. Cell Host Microbe.

[14] Pinto D (2020). Cross-neutralization of SARS-CoV-2 by a human monoclonal SARS-CoV antibody. Nature.

[15] Piccoli L (2020). Mapping neutralizing and immunodominant sites on the SARS-CoV-2 spike receptor-binding domain by structure-guided high-resolution serology. Cell.

[16] Bertoglio F (2021). SARS-CoV-2 neutralizing human recombinant antibodies selected from pre-pandemic healthy donors binding at RBD-ACE2 interface. Nat. Commun..

[17] Zhao J (2020). COVID-19: Coronavirus vaccine development updates. Front. Immunol..

[18] Petherick A (2020). Developing antibody tests for SARS-CoV-2. Lancet.

[19] Wu R (2018). Quantitative and rapid detection of C-reactive protein using quantum dot-based lateral flow test strip. Anal. Chim. Acta.

[20] Gerberding JL, Haynes BF (2021). Vaccine innovations-past and future. N. Engl. J. Med..

[21] Pleskova S, Mikheeva E, Gornostaeva E (2018). Using of quantum dots in biology and medicine. Adv. Exp. Med. Biol..

[22] Amanat F (2020). An in vitro microneutralization assay for SARS-CoV-2 serology and drug screening. Curr. Protoc. Microbiol..

[23] Tan CW (2020). A SARS-CoV-2 surrogate virus neutralization test based on antibody-mediated blockage of ACE2-spike protein-protein interaction. Nat. Biotechnol..

[24] Tong H (2022). Artificial intelligence-assisted colorimetric lateral flow immunoassay for sensitive and quantitative detection of COVID-19 neutralizing antibody. Biosens. Bioelectron..

