# Supplementary material for: Development of a quantum dot-based lateral flow immunoassay strip for rapid and sensitive detection of SARS-CoV-2 neutralizing antibodies
Source: Sci Rep. 2023 Dec 14;13:22253. doi: 10.1038/s41598-023-49244-5 (PMC10721894; doi:10.1038/s41598-023-49244-5)
Supplement: Supplementary file 1 — Supplementary Information. [file 41598_2023_49244_MOESM1_ESM.docx]

**Development of a quantum dot-based lateral flow immunoassay strip for rapid and sensitive detection of SARS-CoV-2 neutralizing antibodies**

Xirong Wang^1＃^, Shulin Shao^2＃^, Huan Ye^4^, Sen Li^4,5^, Bing Gu^1,3＊^&Bo Tang ^1,4,5＊^

^1^ ^Medical Technology School of Xuzhou Medical University, Xuzhou 221004,China^

^2^ ^Department of Laboratory, Nanjing Pukou Hospital of Traditional Chinese Medicine, Nanjing 211800,China^

^3 Laboratory Medicine, Guangdong Provincial People's Hospital. Guangzhou 510000, China^

^4 Nanjing Vazyme Medical Technology Co. Ltd., Nanjing 210046, China^

^5 Nanjing Vazyme Biotechnology Co. Ltd., Nanjing 210046, China^

*Correspondence: Bo Tang.Email:tangbo@vazyme.com;Bing Gu.Email: gubing@gdph.org.cn.

#These authors contributed equally to this work.


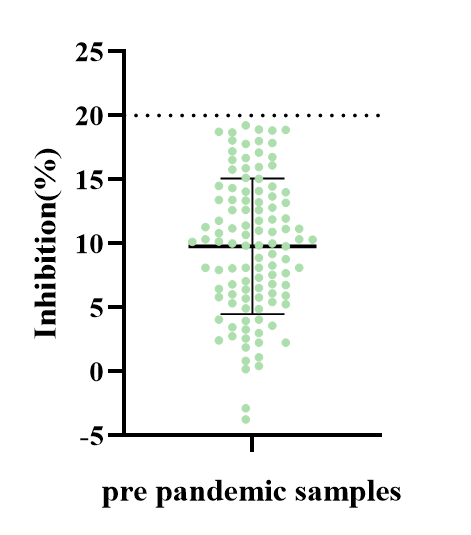


Supplementary Fig 1.ELISA results of pre pandemic samples

Supplementary Table 1 37 ℃ accelerated stability test

Supplementary Table 2 45 ℃ accelerated stability test

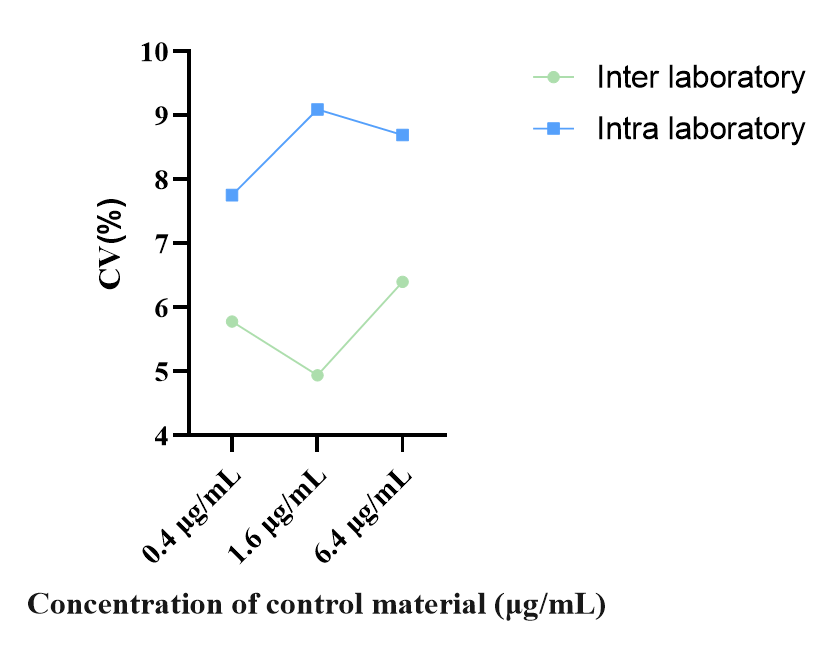


Supplementary Fig 2.Inter and intra laboratory precision of control material
